# Dementia and comorbidities in primary care: a scoping review

**DOI:** 10.1186/s12875-023-02229-9

**Published:** 2023-12-14

**Authors:** Howard Bergman, Soo Borson, Frank Jessen, Pierre Krolak-Salmon, Alessandro Pirani, Jill Rasmussen, Jesus Rodrigo, Daiana Taddeo

**Affiliations:** 1 Department of Family Medicine, McGill University, 5858 Ch. de La Côte-Des-Neiges, Suite 300, Montreal, QC H3S 1Z1 Canada; 2 Department of Family Medicine, Keck School of Medicine, University of Southern California, Los Angeles, USA; 3 Department of Psychiatry, Medical Faculty, University of Cologne, Cologne, Germany; 4 ORPEA Group, Puteaux Cedex, France; 5 Alzheimer’s Association “Francesco Mazzuca”, Cento, Italy; 6 Independent Researcher, Dorking, UK; 7 Spanish Alzheimer’s Confederation (Confederación Española de Alzheimer), Pamplona, Spain; 8 Italian College of General Practitioners and Primary Care (SIMG - Società Italiana Di Medicina Genrale E Cure Primarie), Florence, Italy

**Keywords:** Dementia, Alzheimer’s disease, Primary care, Comorbidities, Person-centred care, Scoping review

## Abstract

**Background:**

People with dementia (PwD) are known to have more chronic conditions compared to those without dementia, which can impact the clinical presentation of dementia, complicate clinical management and reduce overall quality of life. While primary care providers (PCPs) are integral to dementia care, it is currently unclear how PCPs adapt dementia care practices to account for comorbidities. This scoping review maps recent literature that describes the role for PCPs in the prevention, detection/diagnosis and management of dementia in the context of comorbidities, identifies critical knowledge gaps and proposes potential avenues for future research.

**Methods:**

We searched for peer-reviewed literature published between 2017–2022 in MEDLINE, Cochrane Library, and Scopus using key terms related to dementia, primary care, and comorbidity. The literature was screened for relevance by title-abstract screening and subsequent full-text screening. The prioritized papers were categorized as either ‘Risk Assessment and Prevention’, ‘Screening, Detection, and Diagnosis’ or ‘Management’ and were further labelled as either ‘Tools and Technologies’, ‘Recommendations for Clinical Practice’ or ‘Programs and Initiatives’.

**Results:**

We identified 1,058 unique records in our search and respectively excluded 800 and 230 publications during title-abstract and full-text screening. Twenty-eight articles were included in our review, where ~ 50% describe the development and testing of tools and technologies that use pre-existing conditions to assess dementia risk. Only one publication provides official dementia screening guidelines for PCPs in people with pre-existing conditions. About 30% of the articles discuss managing the care of PwD, where most were anchored around models of multidisciplinary care and mitigating potentially inappropriate prescribing.

**Conclusion:**

To our knowledge, this is the first scoping review that examines the role for PCPs in the prevention, detection/diagnosis and management of dementia in the context of comorbidities. Given our findings, we recommend that future studies: 1) further validate tools for risk assessment, timely detection and diagnosis that incorporate other health conditions; 2) provide additional guidance into how comorbidities could impact dementia care (including prescribing medication) in primary care settings; 3) incorporate comorbidities into primary care quality indicators for dementia; and 4) explore how to best incorporate dementia and comorbidities into models/frameworks of holistic, person-centred care.

**Supplementary Information:**

The online version contains supplementary material available at 10.1186/s12875-023-02229-9.

## Background

Key aspects of high-quality dementia care include primary and secondary prevention, improved diagnosis (including timely detection and diagnosis, severity staging, and differentiation of dementia subtypes), communication of the diagnosis to the patient and potential care partners, and post-diagnostic care and treatment [1–4]. There is growing recognition of the importance of primary care providers (PCPs) in all of these aspects of care, especially in timely diagnosis [5–12]. For example, a recent survey by the Alzheimer's Association found that 82% of PCPs reported being at the forefront of providing key components of dementia care [13]. Several countries, such as Canada, Italy and the US, have recognized the importance of PCPs in dementia care and have implemented primary care-based dementia care programs built on principles of interdisciplinary care with other healthcare professionals [7, 14–17]. Furthermore, primary care is consistently mentioned as a vital component in the WHO’s guidelines for risk reduction of cognitive impairment and dementia [2]. Importantly, with the advent of disease‐modifying treatments and blood-based biomarkers for Alzheimer’s Disease (AD), PCPs will likely play a crucial role in identifying people in preclinical or early stages of dementia (e.g. mild cognitive impairment [MCI]) where interventions are most likely to be effective [18–20].

Despite the importance of their role, PCPs face several difficulties in providing optimal dementia care, including insufficient training, consultation time and guidelines [5, 13, 21, 22]. Comorbidity, in particular, increases the complexity of care for people with dementia (PwD) in primary care settings [10, 23, 24]. It is well-established that PwD have more chronic conditions compared to people without dementia, with the most common chronic conditions being diabetes, hypertension, depression, stroke, and vision impairment [1, 2, 25–29]. For example, a recent study of primary care healthcare records found that out of ~ 4,000 PwD, ~ 70% had at least two comorbidities and ~ 50% presented with at least three [27]. In addition, the Centers for Medicare & Medicare Service reported that over 95% of Americans with dementia have another comorbidity and over 50% have five or more comorbidities [30]. Comorbidities can lead to increased disability and medication usage, higher rates of hospitalization, extended hospital stays and reduced quality of life for PwD [25–27, 31]. In addition, comorbidities can cause an atypical clinical presentation of dementia, which can complicate dementia diagnoses and management decisions [25, 27, 32]. Moreover, certain conditions, such as diabetes, hypertension, and stroke, may exacerbate the risk of developing and/or the progression of dementia while cardiovascular therapy may reduce the risk of dementia [2, 26, 33–36]. Recently, Krolak-Salmon et al. (2019) proposed a sequential strategy for diagnosing dementia, which also acknowledged the potential impact of comorbidities on facilitating a timely diagnosis [37].

We are unaware of any clear consensus on how PCPs are (or should be) accounting for the potentially complicating impact of comorbidities on all aspects of dementia care. The objective of this scoping review is to map recent publications that describe a role for primary care in the prevention, detection/diagnosis and management of dementia in the context of comorbidities, identify critical knowledge gaps, and propose potential avenues for future research.

## Methods

We leveraged a scoping review methodology for this study. Unlike a systematic review that collates evidence from a narrow range of studies, a scoping review utilizes findings across various research designs to establish a comprehensive map of available evidence to identify knowledge gaps in the field [38]. A scoping review protocol was developed according to the Preferred Reporting Items for Systematic Reviews and Meta-Analyses (PRISMA) Extension for Scoping Reviews (PRISMA-ScR) guidelines [39] (see Additional_file_ 1.docx). The protocol was not published in advance.

### Search strategy

We searched for peer-reviewed literature using the MEDLINE, Cochrane Library, and Scopus databases to capture a broad range of study designs. The most recent search was executed in December 2022. 

The search strategy was adapted from recent scoping reviews on dementia and comorbidities and was anchored around key terms related to dementia, primary care, and comorbidity [23, 29]. We adopted a broad definition of comorbidities as “any distinct additional clinical entity that has existed or that may occur during the clinical course of a patient who has the index disease under study”, hence including terms such as pain and depression (Table 1) [40]. Using MEDLINE, we identified and included relevant Medical Subject Headings (MeSH) terms for dementia, primary care, and comorbidities, which are listed in Table 1. The concepts related to primary care and dementia were searched in combination with either general comorbidities or disease-specific terms (e.g. hypertension, diabetes, stroke, visual impairment, auditory impairment, cardiovascular disease, chronic kidney disease, chronic obstructive pulmonary disease, COVID-19, depression, insomnia, pain), where the search string was connected by Boolean operators (see Additional_file_ 2.docx for more details).

**Table 1:** Search terms included in the search strategy

	**Key Terms**	**Related Terms**
#1	Dementia	Dementia, Alzheimer’s Disease, Alzheimer’s Disease and Related Dementias, behavioral and psychological symptoms of dementia, dementia with Lewy bodies, frontotemporal dementia, mixed dementia, multi-infarct dementia, vascular dementia
#2	Primary Care	general practitioner, family medicine, family practitioner, primary care, primary health care, primary healthcare
#3	General Comorbidity	associated disease, associated disorder, co-existence, co-existing, co-occurring, comorbid, comorbidity, concomitant, other chronic diseases, other medical conditions, long-term conditions, multi-disciplinary, multidisciplinary, multimorbidity, multiple diseases, multiple morbid, polypathology
#4	Hypertension	blood pressure, hemodynamics, hypertension
#5	Diabetes	blood glucose self-monitoring, diabetes
#6	Stroke	cerebrovascular, cerebrovascular accident, cerebrovascular disorders, stroke
#7	Visual Impairment	blindness, cataract, eye diseases, glaucoma, macular degeneration, nystagmus, retinopathy, vision disorders, visually impaired
#8	Auditory impairment	auditory impairment, deafness, hearing loss
#9	Cardiovascular disease	arrhythmias, atrial fibrillation, cardiovascular disease, congestive heart failure, heart failure, vascular stiffness
#10	Chronic kidney disease	albuminuria, chronic kidney disease, dialysis, hemodialysis, kidney-brain axis, kidney failure
#11	Chronic obstructive pulmonary disease (COPD)	chronic airflow obstructions, chronic obstructive airway disease, chronic obstructive lung disease, chronic obstructive pulmonary disease
#12	COVID-19	COVID-19, coronavirus infection, 2019-nCoV infection, SARS-CoV-2, COVID, and coronavirus disease
#13	Depression	antidepressants, depression
#14	Insomnia	insomnia, parasomnia, sleep deprivation, sleep disorder, sleep disordered breathing, sleep disturbance
#15	Pain	pain, pain assessment, pain experience, pain management
#16	Other specific co-morbidities/conditions	dysphagia, eating behaviour, eating difficulties, infections, mealtime difficulty, neuropsychiatric symptoms, swallowing difficulties

### Inclusion and exclusion criteria

We included peer-reviewed quantitative (e.g. observational studies, randomized controlled trials [RCTs], meta-analysis) and qualitative studies (e.g. one-on-one interviews, guidelines). Abstracts, conference reports, books/chapters, case studies, commentaries, editorials, news articles, protocols, and reviews were excluded. As our emphasis was on recent data, particularly those related to tools and technologies that may be ready for implementation in clinical practice, we focused on studies published within the time frame of January 2017 to December 2022 that were available in English. While reviews were excluded from our analyses, we did examine references of previous reviews (e.g. [23, 28]) for any earlier published literature aligned with our research objectives, and we did not find any papers aligned with our inclusion/exclusion criteria [23, 29]. As our research objective is focused on primary care, we excluded studies based in nursing home and long-term care home settings. We searched for studies that describe how tools and technologies, recommendations for clinical practice, and/or programs and initiatives may assist PCPs in delivering dementia care in the context of comorbidities; these categories are defined in the ‘Reporting the Results*’* below. Notably, only studies clearly articulating how primary care is (or should be) accounting for the impact of comorbidities on dementia care were selected for analysis; this could include, for example, a study evaluating the potential impact of other chronic health conditions, such as diabetes, on the timely diagnosis of dementia. In contrast, studies focusing on managing comorbidities in the context of dementia were not analyzed in this report; the latter could include, for example, challenges associated with managing diabetes in the context of dementia.

### Screening and data extraction

All records were retrieved, and exported into Microsoft Excel, where duplicates were removed. Publications were screened in two phases, first by title-abstract screening, and then by full-text screening according to the ‘Inclusion and Exclusion Criteria’ section. 

To pilot the screening strategy, two reviewers independently screened the first 50 extracted titles and abstracts according to their relevance in addressing one or more of the research objectives. The reviewers compared and discussed discrepancies, and revised the screening strategy accordingly. Using the refined screening strategy, two reviewers independently conducted the title-abstract and full-text screening. Key information was charted from each publication deemed eligible after the screening. These data points included general publication information (e.g. authors, publication year), study characteristics (e.g. study methodology, care setting, geographical location), and comorbid condition(s) influencing dementia care. 

### Reporting the results

The prioritized articles were analyzed qualitatively, and data were presented in a narrative format. Publications were categorized under three headings related to dementia care: ‘Risk Assessment and Prevention’, ‘Screening, Detection, and Diagnosis’ and ‘Management’. Within each heading, the results were further categorized as ‘Tools and Technologies’, ‘Recommendations for Clinical Practice’ and ‘Programs and Initiatives’ where applicable. ‘Tools and Technologies’ included any study describing the identification of comorbidities as risk factors for dementia, risk scores that utilize weighted risk factors to predict the likelihood of developing dementia, prediction tools that translate risk scores into practical tools for primary care, and case management tools. ‘Recommendations for Clinical Practice’ included reports that clearly articulated a clinical practice recommendation for PCPs in delivering dementia care that may be complicated by coexisting comorbidities (e.g. manage chronic illnesses to lower dementia risk, mitigate potentially inappropriate prescribing [PIP]). ‘Programs and Initiatives’ was defined as any report that described a real-world application of a program or initiative (e.g. integrated memory care clinics).

## Results

### Overview of the studies

**Fig. 1:**
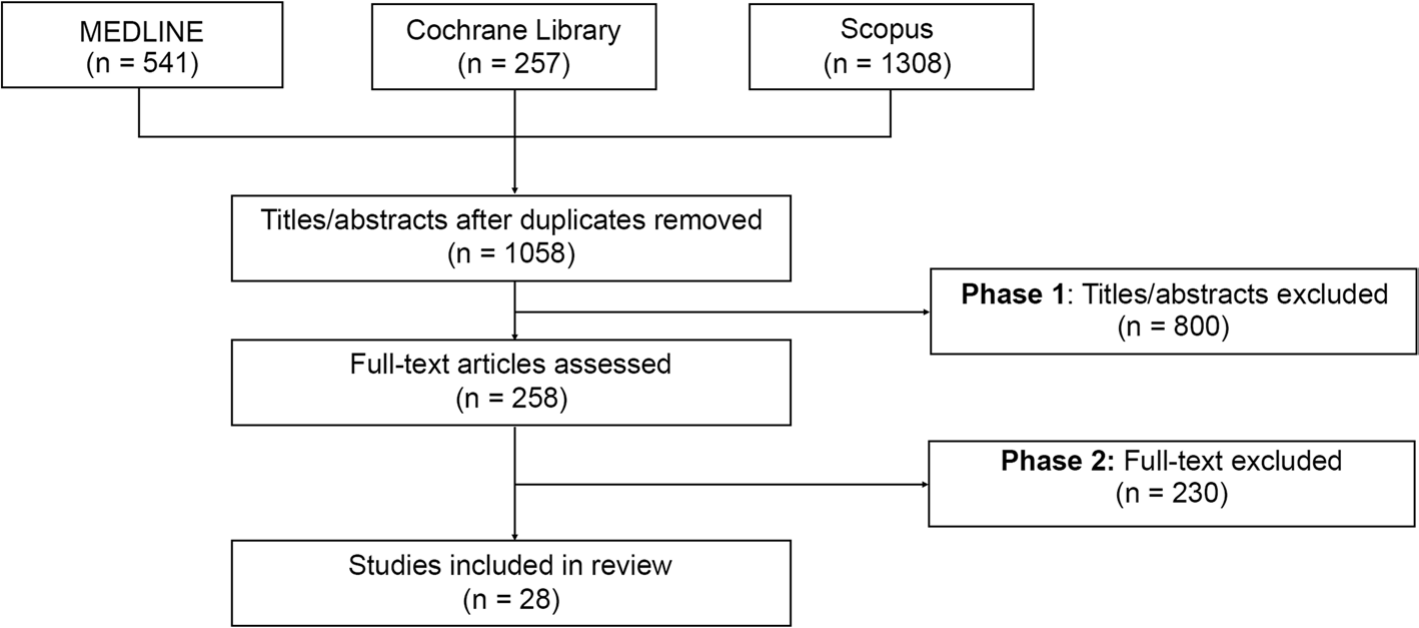
Flow chart of study selection and screening process. A total of 1,058 unique records were identified using our initial search protocol. Following the application of our inclusion and exclusion criteria, 800 publications were removed during the title-abstract screening phase, and an additional 230 publications were removed in the full-text screening phase. The remaining 28 articles were included in the review

A total of 1,058 unique records were identified using our initial search protocol. Following the application of our inclusion and exclusion criteria, 800 publications were removed during the title-abstract screening phase, and an additional 230 publications were removed in the full-text screening phase (Fig. 1). The remaining 28 articles were selected for deeper review (see Additional_file_ 3.xlsx for more details on each article). Characteristics of the 28 articles selected for analysis are listed in Table 2. Most of the articles (*n* = 22) reflected quantitative study designs, including observational studies (*n* = 12) and RCTs (*n* = 3), and six publications were quantitative in nature, which included guidelines (*n* = 2) and interviews (*n* = 2). Most of the articles were focused on individuals with dementia (79%), with the remainder specifying MCI or AD. The most frequent comorbidities noted were hypertension, cardiovascular diseases, diabetes, and depression.

**Table 2:** Characteristics of the 28 studies included for analysis

**Category**	**Details**		**Number of Articles**
**Year of Publication**	2017		5
	2018		5
	2019		2
	2020		4
	2021		7
	2022		5
**Countries Studied**^**a**^	United States of America		9
	United Kingdom		4
	Germany		4
	Singapore		3
	Netherlands		3
	Canada		1
	China		1
	Denmark		1
	Italy		1
	Japan		1
	Portugal		1
	Spain		1
	Taiwan		1
**Type of Study**	Quantitative	Experimental	6
		Meta-Analysis	1
		Observational	12
		Randomized Clinical Trials	3
	Qualitative	Case Study	1
		Guideline	2
		Interviews	2
		Report	1
**Type of Dementia**	Dementia		22
	MCI and dementia		3
	AD		2
	MCI		1
**Comorbidities Studied**^**b**^	Depression		8
	Hypertension		7
	Cardiovascular		7
	General “comorbidities”		7
	Diabetes		6
	Stroke		5
	Auditory Impairment		4
	Other (e.g. dyslipidemia, osteoarticular diseases)		2
	Kidney Disease		1
	Visual Impairment		1
**Categories and Subcategories**	Risk assessment and prevention		15
	Screening, diagnosis, and detection		4
	Management		9
	Tools and technologies		15
	Recommendations for clinical practice		10
	Programs and initiatives		3

^a^Some articles are representative of data from multiple countries

^b^Some articles focus on multiple comorbidities

Our primary research objective was to broadly identify articles that describe the role of primary care in the prevention, detection/diagnosis and management of dementia in the context of comorbidities or other chronic health conditions. The results below are therefore presented under three major headings: ‘Risk Assessment and Prevention’, ‘Screening, Detection, and Diagnosis’ and ‘ Management’. Within each heading, we further categorized findings under the subheadings of ‘Tools and Technologies’, ‘Recommendations for Clinical Practice’ and ‘Programs and Initiatives’ where applicable (see Methods for additional details). Notably, almost half of the 28 prioritized articles (*n* = 13) were focused on tools/technologies in the context of dementia risk assessments and/or prevention. All analyses below are qualitative and are presented in narrative format.

### Risk assessment and prevention

#### Tools and technologies

Twelve articles describe tools and technologies for risk assessments and prevention. The majority of these publications either identified predictive factors for dementia (e.g. diabetes, hypertension, subjective short-term memory difficulties, and right-sided hearing loss) or described the development/validation of risk prediction scores/models that could potentially be used in primary care settings. Below we summarize key articles describing tools and technologies in more advanced stages of development (see Table 3 for a description of all identified reports).

**Table 3:** Summary of tools and technologies to support PCPs in providing dementia care that considers comorbidities

**Stage of Patient Pathway**	**Dementia Type**	**Tools & Technologies**	**Description**	**Stage of Development**	**Citation**
**Risk Assessment and Prevention**	Dementia	Dementia risk prediction metrics	This meta-analysis identified factors (e.g. depression, history of stroke and orthostatic hypotension, diabetes) that are consistently associated with dementia, which should be further explored when developing a dementia risk prediction tool for primary care for PCPs to implement early interventions to slow the progression of dementia	Predictors were selected for a dementia risk prediction tool; the tool was not developed	[41]
	Cognitive decline in MCI	Dementia risk prediction metrics	This randomized clinical trial (RCT) conducted in China with a cohort of individuals aged 55–65 found that right-sided hearing loss may be a predictor of cognitive decline in older adults. This morbidity can be utilized as a marker to support PCPs in identifying high-risk individuals for early prevention of cognitive decline	Risk predictor suggested; the tool was not developed	[42]
	Dementia	Dementia risk prediction metrics	This observational study conducted in Portugal identified gastrointestinal diseases as potential risk factors when screening for dementia in a younger cohort aged 50 and older, which could be readily implemented in primary care. However, gastrointestinal diseases are only demonstrated to be highly correlated with dementia risk when used in a multivariate model with other factors such as gender, age, APOE ε4 allele, female gender, and low education, and needs to be further validated	Risk predictor suggested; the tool was not developed	[43]
	Dementia	MyHealthNetwork	MyHealthNetwork is a digital health tool to monitor brain health to lower dementia risk, especially given the positive association between hypertension and the risk of future dementia. The tool is designed for patients to record their blood pressure readings and readily share the data with their PCPs to monitor and detect hypertension and ultimately potential changes to brain health. Future developments of this platform will capture additional metrics related to brain health (e.g. blood glucose level, sleep patterns) to provide more comprehensive insights into one’s brain health	Prototype development	[44]
	Dementia	Dementia prediction model	This multivariable risk prediction model incorporates metrics easily collected in the primary care setting (e.g. age, sex, hypertension, education level, diabetes, body mass index (BMI), history of stroke, smoking status, and sedentariness) to identify individuals at high risk of developing dementia within 10 years of the assessment. Assessment results can guide PCPs in providing risk factor-based interventions to lower the risk of future dementia	Developed with a sample of 795 participants aged 65 +	[45]
	AD	Zaragoza Dementia and Depression Project (ZARADEMP) Alzheimer Dementia Risk Score	The ZARADEMP risk score incorporates socio-demographic (age, sex, marital status, educational level), psychological (depression and anxiety), behavioral (obesity and alcohol and tobacco use), and medical (diabetes, hypertension, stroke, acute myocardial infarction, history of angina, and hearing loss) factors to predict the risk of developing AD within 5 years of the assessment. This score can identify at-risk individuals in primary care settings and implement preventative and therapeutic strategies in earlier disease stages	Developed with a sample of 3044 participants aged 65 + , not yet validated	[46]
	MCI and dementia	Singapore Longitudinal Ageing Study (SLAS) MCI risk prediction index	The SLAS risk index incorporates 20 indicators commonly measured in primary care settings, including psychosocial (e.g. life satisfaction, living alone), lifestyle (e.g. education level, frequency of physical and social activities), and health risk factors (e.g. sex, age, smoking history and status, cardio-metabolic and vascular risk factors) to predict the risk of MCI or dementia within 4.5 years of the assessment. This assessment is to identify individuals in community settings at the pre-dementia stage for preventative interventions	Validated with a sample of ~ 4374 participants aged 55 +	[47]
	Dementia	The Rapid Assessment of Dementia Risk (RaDaR)	The RADaR score uses predictors capturing demographic factors (e.g. sex, age), clinical predictors (e.g. blood pressure, stroke, medications), medical history (e.g. cancer, smoking, hypertension, diabetes), memory complaints, functional disability, psychological factors, cognitive testing, and motor evaluations to measure the risk of developing dementia within 3 years of the assessment for individuals over the age of 65 years. This tool can be utilized in primary care to identify screening frequency for high-risk individuals	Validated with a sample of 1308 participants (average age of 65 +)	[48]
	Dementia	Dementia risk prediction model	This multivariate dementia risk prediction model used predictors such as sex, age, education level, history of diabetes, systolic blood pressure, history of stroke, smoking status, parental history of dementia, and presence of depressive symptoms to predict the risk of developing dementia within 10 years of the assessment to identify individuals in primary care to be referred to specialized care settings for diagnosis and to stratify high-risk individuals to inform clinical trial design targeting early dementia interventions	Validated with a sample of 742 participants aged 60 +	[49]
	AD	AD prediction score	The AD prediction score is to be used in primary care to classify patients who will develop AD within 15 years of the evaluation. The prediction score incorporates the symptomatic and drug use predictors, including the presence of memory disorders, hallucinations, anxiety, and depression, and the use of non-steroidal anti-inflammatory drugs. This score can allow PCPs to screen for at-risk individuals in primary care settings to implement preventative and therapeutic strategies	Validated with a sample of 66,659 participants aged 60 +	[50]
	MCI	mCAIDE	The modified Cardiovascular Risk Factors, Aging, and Dementia (mCAIDE) score includes easily assessed and self-reported metrics (e.g. sex, age, education, history of cholesterol), non-invasive clinical indicators of chronic illness (e.g. systolic blood pressure), and physical performance-based indicators (e.g. mini Physical Performance Testing [mPPT]), all which can be readily implemented in the community and primary care to screen for cognitive impairment and identify at-risk patients to target for interventions to delay dementia progression	Validated with a sample of 219 participants aged 65 +	[51]
	Dementia	Anticholinergic Cognitive Burden Scale (ACB)	The compounded use of anticholinergic medications in older adults for chronic conditions (e.g. cardiovascular drugs, anti-histamines, drugs affecting the nervous system) has increased the vulnerability of the patients to anticholinergic adverse events, such as cognitive impairment which may develop into dementia. The use of ACB in primary care can minimize suboptimal prescribing and choose medications to reduce the anticholinergic burden and hence lower dementia risk	An analysis of 116,043 older adults demonstrated the effectiveness of the ACB scale at predicting the risk of incidence dementia in individuals aged 65–84; the tool can be used to guide PCP prescriptions	[52]
**Screening, Detection, and Diagnosis**	MCI, dementia	Diagnostic algorithm	This diagnostic algorithm assists PCPs in choosing the most suitable cognitive test (e.g. clock drawing test, MoCA, MMSE) per suspected case of cognitive impairment, which depends on the likelihood of the cognitive impairment after history taking and an informant interview. The algorithm considers other chronic conditions (e.g. depression, diabetes, cardiovascular factors) as risk factors when determining whether or not cognitive impairment is a potential diagnosis before selecting the appropriate cognitive test	Framework for algorithm is proposed, but not yet developed	[53]
**Management**	Dementia	CASEPLUS-SimPat	CASEPLUS-SimPat is a web-based case management system that allows different healthcare professionals (e.g. PCPs and hospital employees) to access patient data to coordinate treatment, which will be particularly valuable for PwD with multimorbidity as they will require more complex care situations	Developed, but not yet validated	[54]

Two studies describe the development of risk scores using medical conditions and other metrics as risk factors that are assessable in primary care. These include a multivariable risk model that claims to predict future dementia [45], and the Zaragoza Dementia and Depression Project (ZARADEMP) Alzheimer Dementia Risk Score that aims to predict the risk of developing AD [46]. Both studies used development cohorts aged 65 and older, and their prediction scores may not be applicable in assessing risk in younger populations.

Five studies describe developed and validated dementia prediction models which incorporate risk factors, such as medical conditions. One example is the Singapore Longitudinal Ageing Study (SLAS) MCI risk prediction index that incorporates both demographic factors (e.g. age, sex, education level) and medical conditions (e.g. auditory impairment and cardio-metabolic risk factors) to predict MCI and dementia in the community setting, which was validated with a cohort of 4,374 individuals in Singapore [47]. The remaining studies describe the development and validation of risk prediction models for dementia (e.g. Rapid Assessment of Dementia Risk [RaDaR]; Licher and colleagues’ dementia risk prediction model; the modified Cardiovascular Risk Factors, Aging, and Dementia [mCAIDE] score) [48, 49, 51] and AD (e.g. the AD prediction score) [50], which all incorporate medical conditions, such as depression and diabetes. These models were developed for PCPs to identify those at high risk of developing MCI and/or dementia and to initiate interventions or advise on lifestyle modifications to lower potential dementia risk.

One observational study conducted in Taiwan describes the implementation of a previously developed tool—the Anticholinergic Cognitive Burden Scale (ACB)— for older adults in primary care settings. Use of anticholinergics may result in adverse effects such as cognitive impairment, and inherently increase the risk of dementia. This study found that the ACB scale was more effective in comparison to other anticholinergic burden scoring systems in assisting PCPs to optimize anticholinergic medication in individuals with comorbidities to lower the risk of dementia [52].

Finally, one report describes a digital tool—the MyHealthNetwork—which may assist PCPs in monitoring blood pressure for timely detection and treatment of hypertension to lower dementia risk in a relatively younger population (aged 45 and over) [44]. However, MyHealthNetwork is in the early stages of development with plans to further expand the platform to include other health risk factors, such as blood glucose levels and sleep activity for a more comprehensive assessment [44].

#### Recommendations for clinical practice

Three articles provide clinical practice recommendations for PCPs related to risk assessment/prevention. This includes an official guideline from the Canadian Consensus Conference on Diagnosis and Treatment of Dementia (CCCDTD) for primary care physicians and specialized clinics to assess dementia risk in older adults. The guideline includes a component recommending that PCPs be aware of non-cognitive dementia risk factors such as neuro-behavioural, motor, sleep, sensory, and frailty variables to assess dementia risk in older adults [55]. The remaining two articles include observational studies focused on managing chronic illnesses in primary care to decrease dementia risk. One study was conducted in the US and found that the control of blood pressure using antihypertensives can prevent the onset of dementia in older adults. However, the findings are limited to the African American demographic who are 70 years and older [56]. The second study was conducted in Germany and found that subjective cognitive decline in short-term memory, as assessed by PCPs, predicted the risk of vascular and all-cause dementia in a community-based cohort of adults aged 50–75 [57]. These findings highlight the importance of identifying this high-risk population in primary care for early intervention for cardiovascular diseases to prevent or delay cognitive decline [57].

### Screening, detection, and diagnosis

#### Tools and technologies

One recent study from the Netherlands describes an algorithm to support PCPs in choosing the most suitable cognitive test (e.g. clock drawing test, Montreal Cognitive Assessment [MoCA], Mini Mental State Examination [MMSE]) when detecting cognitive impairment (additional details in Table 3) [53]. This algorithm circumvents a “one size fits all” approach, as the framework considers existing chronic conditions as risk factors, providing a more holistic approach to detecting MCI and/or dementia [53]. However, this algorithm has not yet been validated and is not ready for clinical implementation [53].

#### Recommendations for clinical practice

Three studies provide recommendations to improve the timely diagnosis of dementia in primary care settings in individuals with coexisting conditions associated with increased risk of dementia. One of these articles describes a guideline—the American Diabetes Association’s (ADA’s) Standards of Medical Care in Diabetes—which recommends annual dementia screening for individuals aged 65 and older who have diabetes, as diabetes is associated with an increased risk of dementia [58]. Two studies evaluated the benefits of preventative dementia screening for older adults in primary care. In a qualitative interview-based study conducted in England, clinicians highlighted the benefits of cognitive screening in post-stroke survivors in primary and secondary care settings despite the absence of guidelines advocating for preventative cognitive screening in this population [59]. In contrast, an RCT conducted in the US found that screening of Alzheimer’s Disease and Related Dementias (ADRDs) in older adults, including those with comorbidities such as chronic lung disease and diabetes, was neither harmful nor beneficial towards one’s health-related quality of life [60].

### Management

#### Tools & technologies

One study in Germany developed and conducted a preliminary evaluation of a web-based case management system called CASEPLUS-SimPat to facilitate and coordinate care between PCPs, healthcare professionals, and hospital employees specifically for multimorbid dementia patients [54]. The tool was piloted in a hospital setting and did not include PCPs, and therefore will require further studies to validate the feasibility and effectiveness of CASEPLUS-SimPat in primary care settings [53].

#### Recommendations for clinical practice

Three articles provide recommendations for PCPs in managing dementia in the context of comorbidities through pharmacological (*n* = 2) and non-pharmacological management (*n* = 1). Two studies provide recommendations to mitigate potentially inappropriate prescribing and consequential adverse health outcomes in PwD due to coexisting chronic condition(s). One observational study conducted in the UK identified that the prevalence of PIP was the highest in PwD with comorbidities, such as severe mental illness and depression, in primary care [61]. The most common PIP criteria were therapeutic duplication and the prescription of anticholinergic drugs, where anticholinergics may be prescribed for both dementia symptoms as well as other comorbidities (e.g. cardiovascular diseases) [61]. An RCT conducted in Germany also identified high PIP of anticholinergic antidepressants and long-acting benzodiazepines in PwD, which are often used to treat behavioural symptoms of dementia as well as coexisting depression, anxiety, or insomnia [62]. While authors of these studies stress that more evidence and research are required to guide deprescribing for coexisting chronic conditions in PwD, PCPs are recommended to conduct medication reviews using approaches such as the STOPP/START criteria, the Potentially Inappropriate Medications in the Elderly (PRISCUS) list, common PIP criteria (e.g. therapeutic duplication, prescription of anticholinergic drugs), and/or focus on medications for comorbidities with the greatest risk of PIP (e.g. depression and cardiovascular disease) [61, 62]. One article reports non-pharmacological management for PwD with insomnia and disturbed sleep patterns, where PCPs can address chronic health conditions (e.g. respiratory disease, chronic pain, anxiety) that may affect sleep quality [63].

Two articles provide PCP recommendations to enhance the quality of care for PwD with comorbidities. One observational study conducted in the UK found that care continuity of PwD with PCPs reduced unnecessary transitions to hospital settings in the last 90 days of life, particularly among home-dwelling PwD with comorbidities. Based on these findings, the authors of the study recommended for PCPs to identify PwD who require a palliative care approach using tools such as the Supportive and Palliative Care Indicators Tool, the Electronic Frailty Index, and the Integrated Palliative Care Outcome Scale for People with Dementia (IPOS-Dem) [64]. Another study from Singapore recommends that PCPs refer PwD to memory clinics after diagnosis to provide a multidisciplinary approach to care. At the memory clinic, a multidisciplinary team comprised of clinicians, dementia-trained nurses, physiotherapists, social workers, occupational therapists, and psychologists can consult on comorbidities in addition to managing dementia [65].

#### Programs and initiatives

Two observational studies explore the effectiveness of multidisciplinary teams in Integrated Memory Care Clinics (IMCC) which provides coordinated primary care directed by advanced practice registered nurses (APRNs) from different disciplines (e.g. neurology, geriatric psychiatry, and palliative care) and partners with caregivers and PwD (including those with comorbidities) to deliver patient-centred care [66]. These studies were conducted in the US and report preliminary data on the sustainability and success of IMCC implementation as measured through cost reductions to the health care system, positive caregiver experience, and improvement of neuropsychiatric symptoms in PwD with underlying comorbidities, such as diabetes, hypertension, and hyperlipidemia [66, 67]. However, considering the small sample size of these studies, additional validation will be required to understand the effectiveness of IMCCs to the broader PwD population [66, 67].

One study describes the development and successful implementation of the Cognition and Mobility Care Management (CMCM) primary care program in the US, where nurse care managers collaborate with PCPs to comprehensively design tailored behavioral, psychosocial, and medical interventions for older adults with dementia and falls [68]. While the qualitative study found that this program was well received by patients and caregivers, it should be noted that this study was conducted in a cohort of Latino and African American ethnicities in underserved communities and will need to be validated in the broader population for large-scale implementation [68].

## Discussion

The primary objective of this review was to identify tools/technologies, recommendations for clinical practice, and programs and initiatives that may support or enhance the role of primary care in the prevention, detection/diagnosis and management of dementia in the context of comorbidities. Our findings above are organized under three major headings across the dementia pathway: ‘Risk Assessment and Prevention’, ‘Screening, Detection, and Diagnosis’ and ‘ Management’. Notably, almost half of the prioritized articles were focused on tools/technologies in the context of dementia risk assessments and/or prevention. The majority of these reports either identified predictive factors for dementia or described the development/validation of risk prediction scores or models that could potentially be used in primary care settings (see Table 3). In contrast, only a handful of reports that were identified focused on ‘Screening, Detection, and Diagnosis’ or ‘ Management’. The main emphasis of the latter was on topics related to timely diagnosis, descriptive models of (or tools to enable) multidisciplinary care, and pharmacological and non-pharmacological management of dementia in the context of comorbidities. Below we summarize key takeaways from these results, identify limitations and provide some recommendations for future research.

As noted above there is a growing role for primary care in dementia prevention, risk assessments, timely detection and diagnoses of dementia, but PCPs face challenges in fulfilling this role, especially when other health conditions are present (see Introduction). In our review, 68% of the included papers were focused on aspects related to either risk assessments or diagnosis. 13 of the articles we identified were focused on tools that have incorporated comorbidities in risk assessments and screening. Encouragingly, many of these are showing promising results, such as a multivariable risk model that aims to predict the development of dementia [39], and the ZARADEMP Alzheimer Dementia Risk Score that aims to predict the risk of developing AD [46] (see Table 3). However, most tools are in the early stages of development, including discovering predictors to incorporate into the tools, developing a prototype, and validating the tools in larger cohorts [41–51] (see Table 3). Moreover, there is a need for greater guidance and standardization to determine which tools should be used depending on the patient’s unique situation, including their socioeconomic status, the type of dementia and the presence of other chronic conditions [69, 70]. For example, we only found two studies that accounted for comorbidities in the risk of young onset dementia (e.g. individuals under the age of 65) and only one study examined screening for dementia in underserved communities [43, 57, 68].

These tools and technologies could strengthen the role of PCPs in identifying high risk populations for dementia and support a secondary prevention strategy. In addition to enhancing the risk of developing dementia, there is some evidence that the control of comorbidities could also support a secondary prevention strategy; for example, a meta-analysis of cohort studies has reported that individuals with diabetes who were taking metformin were less likely to develop cognitive impairment compared to those without treatment or taking other medications [71]. Interestingly, we identified one study that found that controlling blood pressure with antihypertensives can prevent the onset of dementia in older adults [56]. However, we did not find any other prescriptive guidelines within the last 5 years (see study limitations below) apart from those in the CCCDTD, which recommended PCPs to be aware of non-cognitive dementia risk factors, including comorbidities, to assess dementia risk in older adults [55]. Greater guidance into how all aspects of dementia care (e.g. prevention, risk assessment, screening, detection, diagnosis, and care management) in the primary care setting need to be adapted in the context of comorbidities.

Only nine publications focused on adapting dementia management in primary care to account for comorbidities. These papers were either focused on recommendations to mitigate PIP (with respect to anticholinergics and therapeutic duplication) or enhance care quality, or described programs/initiatives to provide multidisciplinary care for PwD. In terms of PIP, our review identified two studies that recommended medication reviews by PCPs to include approaches such as the STOPP/START criteria, the PRISCUS list, common PIP criteria, and/or focus on medications for comorbidities with the greatest risk of PIP (e.g. depression and cardiovascular disease) [54, 60]. As these studies noted, there is a need for additional studies to guide prescribing for coexisting chronic conditions in PwD.

There is a growing literature on primary care quality indicators for chronic conditions including dementia. For example, Sourial et al. (2022) developed a framework of primary care quality indicators adapted to PwD; however, these indicators do not appear to account for the influence of chronic conditions on dementia care [72]. Similarly, Bodenheimer et al. (2002) described a chronic care model that is not specific to dementia but helps to guide higher-quality chronic illness management within primary care [73]. Notably, none of the publications included in our review described indicators of high-quality dementia care that account for the impact of comorbidities, and we recommend that future studies reflect on how to incorporate comorbidities into these primary care indicators for dementia.

Finally, there is a growing literature base describing the importance of holistic, person-centred dementia care that incorporates a patient’s overall health (including their comorbidities), well-being and values. It has been noted that PCPs are well-positioned to provide such care [1, 11, 23]. In our review, two studies recommended that PCPs account for comorbidities when delivering person-centred dementia care [63, 65]. In addition, we also identified two programs/initiatives (e.g. IMCC and CMCM) that aim to incorporate principles of collaborative, interdisciplinary, person-centred dementia care that considers an individual’s comorbidities. However, these programs are in the early stages of implementation and further validation is required to determine applicability to the broader PwD population. Further exploration of the role of comorbidities in such frameworks/models and their applicability in primary care would be beneficial.

At least two previous reports have reviewed the literature on comorbidities and dementia more broadly. For example, Bunn et al. [28]  found that there was limited information on healthcare providers’ experiences of managing people living with dementia and comorbid health conditions and how the presence of dementia influences care [29]. Moreover, more recently, Dunn et al. (2022) found that current definitions of comorbidity and multimorbidity are heterogeneous, reductionist and disease-focused, and fail to accurately reflect the health and well-being of people with dementia [23]. The authors of this study rightfully note that PCPs, in particular, could bring a more holistic, person-centred approach to dementia care by focusing on the overall health of the individual, including their comorbidities. Indeed, this viewpoint is well-aligned with the WHO’s recent publication on a primary healthcare measurement framework and indicators, which underscores the role of PCPs in providing continuous, comprehensive and person-centred care to individuals and families [74].

Given the growing recognition of the importance of PCPs in all aspects of dementia care, a major objective of this review was to identify tools/technologies, guidelines or programs/initiatives that could support PCPs in accounting for the potentially complicating impact of comorbidities on dementia care. Given the highly focused scope of this review, we therefore explicitly excluded articles which focused on the management of comorbidities in the presence of dementia, and instead focused our efforts on those that discussed the management of dementia in the presence of comorbidities (see Methods). While we believe that this approach is reasonable from a methodological and analytical perspective, it nonetheless results in some conceptual limitations from a clinical perspective. For example, in the results above, we outline reports that recommend a medication review for PIP for dementia (e.g. for anticholinergics); however, we did not discuss the clinically competing demand for appropriate management of prescribing needs for comorbidities, which is a key element of a medication review [75]. More generally, as described in the above paragraph, PCPs play an integral role in providing holistic person-centred care; consequently, in clinical practice, dementia must not be viewed as a siloed entity that needs to be managed at the exclusion of comorbidity management.

This study has other technical limitations. Our search was limited to peer-reviewed articles that were written in English, and had the keywords “dementia” and/or “Alzheimer”. Grey literature was not included in our search. Although our search parameters did not include geographic criteria, there was a large representation from Europe (*n* = 15) and the USA (*n* = 9), which may influence our findings.

Notwithstanding these limitations, to our knowledge, this is the first scoping review that examines the role for primary care in the management of dementia in the context of comorbidities and we believe that our findings add to the growing body of literature in this area and could inform future studies to close knowledge gaps (see Conclusions below).

## Conclusions

Over 65% of the papers included in our review focused on risk assessments and prevention with a majority describing dementia assessment tools that incorporate comorbidities. Furthermore, we only found a handful of studies that describe how post-diagnostic dementia care in the primary care setting needs to account for the impact of comorbidities. Given our findings, we recommend that future studies: 1) further validate tools for risk assessment, timely detection and diagnosis that incorporate other health conditions; 2) provide additional guidance into how comorbidities could impact dementia care practice and secondary prevention (including prescribing medication) in primary care settings; 3) incorporate comorbidities into primary care quality indicators for dementia; and 4) explore how to best incorporate both dementia and comorbidities into models/frameworks of holistic, person-centred care.

### Supplementary Information

**Additional file 1.** Preferred Reporting Items for Systematic Reviews and Meta-Analyses extension for Scoping Reviews (PRISMA-ScR) Checklist.

**Additional file 2.** Search strings used in MEDLINE according to Key Terms defined in Table 1.

**Additional file 3.** Characteristics of each source of evidence included in this scoping review, and the relevant data extracted from each study.

## Data Availability

Additional_file_ 1.docx: Preferred Reporting Items for Systematic Reviews and Meta-Analyses extension for Scoping Reviews (PRISMA-ScR) Checklist. Additional_file_ 2.docx: Search strings used in MEDLINE according to Key Terms defined in Table 1. Additional_file_ 3.xlsx: Characteristics of each source of evidence included in this scoping review, and the relevant data extracted from each study.

## Abbreviations

ACB: Anticholinergic Cognitive Burden Scale
AD: Alzheimer’s Disease
ADA: American Diabetes Association
ADRD: Alzheimer’s Disease and Related Dementias
APRN: Advanced Practice Registered Nurse
BMI: Body Mass Index
CCCDTD: Canadian Consensus Conference on Diagnosis and Treatment of Dementia
CMCM: Cognition and Mobility Care Management
IMCC: Integrated Memory Care Clinics
mCAIDE: Modified Cardiovascular Risk Factors, Aging, and Dementia
MCI: Mild Cognitive Impairment
MMSE: Mini Mental State Examination
MoCA: Montreal Cognitive Assessment
mPPT: Mini Physical Performance Testing
PCPs: Primary Care Providers
PIP: Potentially Inappropriate Prescribing
PRISCUS: Potentially Inappropriate Medications in the Elderly
PRISMA-ScR: Preferred Reporting Items for Systematic Reviews and Meta-Analyses Extension for Scoping Reviews
PwD: People with Dementia
RaDaR: Rapid Assessment of Dementia Risk
RCTs: Randomized Control Trials
SLAS: Singapore Longitudinal Ageing Study
ZARADEMP: Zaragoza Dementia and Depression Project
