# Supplementary material for: Dementia and comorbidities in primary care: a scoping review
Source: BMC Prim Care. 2023 Dec 14;24:277. doi: 10.1186/s12875-023-02229-9 (PMC10720181; doi:10.1186/s12875-023-02229-9)
Supplement: Supplementary file 2 — Additional file 2. Search strings used in MEDLINE according to Key Terms defined in Table 1. [file 12875_2023_2229_MOESM2_ESM.docx]

**Additional File 2:** Search strings used in MEDLINE according to Key Terms defined in Table 1.

| **Search Components** | **MEDLINE Search Query** |
| --- | --- |
| Primary Care, Dementia, General Comorbidity  (Key Terms #1-3) | (general practitioner[tw] OR family medicine[tw] OR family practitioner[tw] OR primary care[tw] OR primary health care[tw] OR primary health-care[tw] OR primary healthcare[tw] OR post-diagnosis care[tw] OR post diagnosis care[tw] OR post-diagnostic care[tw] OR post diagnostic care[tw])  AND  (dement*[tw] OR Alzheimer*[tw])  AND  (associated disease[tw] OR associated disorder[tw] OR co-exist*[tw] OR co-occurring[tw] OR comorbid*[tw] OR concomitant[tw] OR other chronic[tw] OR other cognitive[tw] OR other medical[tw] OR long-term condition[tw] OR long term condition[tw] OR multi-disciplinary teams[tw] OR multidisciplinary teams[tw] OR multimorbidity[tw] OR multiple diseases[tw] OR multiple morbid[tw] OR polypathology[tw])  NOT (Book[Publication Type]) NOT (Case Reports[Publication Type]) NOT (Comment[Publication Type]) NOT (Editorial[Publication Type]) NOT (English Abstract[Publication Type]) NOT (News[Publication Type]) NOT (Newspaper Article[Publication Type]) NOT (Review[Publication Type]) NOT (Case Reports[Publication Type])  Limit to 2017-2022, English |
| Primary Care and Dementia-related and Disease specific terms  (Key Terms #1, 2, 4-16) | (general practitioner[tw] OR family medicine[tw] OR family practitioner[tw] OR primary care[tw] OR primary health care[tw] OR primary health-care[tw] OR primary healthcare[tw] OR post-diagnosis care[tw] OR post diagnosis care[tw] OR post-diagnostic care[tw] OR post diagnostic care[tw])  AND  (dement*[tw] OR Alzheimer*[tw])  AND  ((blood pressure[tw] OR hemodynamic[tw] OR hypertension[tw]) OR (blood glucose self-monitoring[tw] OR blood glucose self monitoring[tw] OR diabet*[tw]) OR (cerebrovascular[tw] OR stroke[tw]) OR (blindness[tw] OR cataract[tw] OR eye disease[tw] OR glaucoma[tw] OR macular degeneration[tw] OR nystagmus[tw] OR retinopathy[tw] OR vision disorders[tw] OR visual impair*[tw]) OR (auditory impairment[tw] OR deafness[tw] OR hearing loss[tw]) OR (arrhythmias[tw] OR atrial fibrillation[tw] OR cardiovascular disease[tw] OR heart failure[tw] OR vascular stiffness[tw]) OR (albuminuria[tw] OR chronic kidney disease[tw] OR CKD[tw] OR dialysis[tw] OR kidney-brain axis[tw] OR kidney failure[tw]) OR (chronic airflow obstruction*[tw] OR COAD[tw] OR chronic obstructive airway disease[tw] OR chronic obstructive lung disease[tw] OR chronic obstructive pulmonary disease[tw] OR COPD[tw]) OR (antidepressant[tw] OR anti-depressant[tw] OR depress*[tw]) OR (insomnia[tw] OR parasomnia[tw] OR sleep deprivation[tw] OR sleep disorder[tw] OR sleep disturbance[tw]) OR (pain[tw]) OR (dysphagia[tw] OR eat*[tw] OR infection*[tw] OR mealtime difficult*[tw] OR neuropsychiatric symptom[tw] OR swallowing difficult*[tw]))  NOT (Book[Publication Type]) NOT (Case Reports[Publication Type]) NOT (Comment[Publication Type]) NOT (Editorial[Publication Type]) NOT (English Abstract[Publication Type]) NOT (News[Publication Type]) NOT (Newspaper Article[Publication Type]) NOT (Review[Publication Type]) NOT (Case Reports[Publication Type])  Limit to 2017-2022, English |
